# Lesion Localization and Pathological Diagnosis of Ovine Pulmonary Adenocarcinoma Based on MASK R-CNN

**DOI:** 10.3390/ani14172488

**Published:** 2024-08-27

**Authors:** Sixu Chen, Pei Zhang, Xujie Duan, Anyu Bao, Buyu Wang, Yufei Zhang, Huiping Li, Liang Zhang, Shuying Liu

**Affiliations:** 1 College of Veterinary Medicine, Inner Mongolia Agricultural University, Zhao Wu Da Road No. 306, Hohhot 010018, China; chans1001@163.com (S.C.); 15149366412@163.com (P.Z.); duanxujie1010@163.com (X.D.); 15848106411@163.com (A.B.); enjoy_zyf@163.com (Y.Z.); lihuiping16300000@163.com (H.L.); zhangliang88_2006@126.com (L.Z.); 2 Inner Mongolia Key Laboratory of Basic Veterinary Science, Hohhot 010018, China; 3 Key Laboratory of Clinical Diagnosis and Treatment Technology in Animal Disease, Ministry of Agriculture, Hohhot 010018, China; 4 College of Computer and Information Engineering, Inner Mongolia Agricultural University, Hohhot 010010, China; bywang08@imau.edu.cn

**Keywords:** artificial intelligence, deep learning, instance segmentation, MASK R-CNN, ovine pulmonary adenocarcinoma, ovine pulmonary adenocarcinoma diagnosis, pathological diagnosis

## Abstract

**Simple Summary:**

In this study, a Common Objects in Context dataset of ovine pulmonary adenocarcinoma pathological images was constructed based on 7167 annotated typical lesions from 61,063 lung pathological images of ovine pulmonary adenocarcinoma. This study aimed to develop a mask regional convolutional neural network model for the localization and pathological diagnosis of ovine pulmonary adenocarcinoma lesions. The model achieved a mean average specificity of 0.573 and an average sensitivity of 0.745, with consistency rates of 100% for junior pathologists and 96.5% for senior pathologists in the diagnosis of ovine pulmonary adenocarcinoma. The successful development of this model not only facilitates the rapid diagnosis of ovine pulmonary adenocarcinoma by different personnel in practical applications but also lays a foundation for the transition from traditional pathology to digital pathology in the livestock industry.

**Abstract:**

Ovine pulmonary adenocarcinoma (OPA) is a contagious lung tumour caused by the Jaagsiekte Sheep Retrovirus (JSRV). Histopathological diagnosis is the gold standard for OPA diagnosis. However, interpretation of traditional pathology images is complex and operator dependent. The mask regional convolutional neural network (Mask R-CNN) has emerged as a valuable tool in pathological diagnosis. This study utilized 54 typical OPA whole slide images (WSI) to extract 7167 typical lesion images containing OPA to construct a Common Objects in Context (COCO) dataset for OPA pathological images. The dataset was categorized into training and test sets (8:2 ratio) for model training and validation. Mean average specificity (mASp) and average sensitivity (ASe) were used to evaluate model performance. Six WSI-level pathological images (three OPA and three non-OPA images), not included in the dataset, were used for anti-peeking model validation. A random selection of 500 images, not included in the dataset establishment, was used to compare the performance of the model with assessment by pathologists. Accuracy, sensitivity, specificity, and concordance rate were evaluated. The model achieved a mASp of 0.573 and an ASe of 0.745, demonstrating effective lesion detection and alignment with expert annotation. In Anti-Peeking verification, the model showed good performance in locating OPA lesions and distinguished OPA from non-OPA pathological images. In the random 500-image diagnosis, the model achieved 92.8% accuracy, 100% sensitivity, and 88% specificity. The agreement rates between junior and senior pathologists were 100% and 96.5%, respectively. In conclusion, the Mask R-CNN-based OPA diagnostic model developed for OPA facilitates rapid and accurate diagnosis in practical applications.

## 1. Introduction

Ovine pulmonary adenocarcinoma (OPA) is an infectious lung tumour caused by Jaagsiekte Sheep Retrovirus (JSRV) [1]. OPA has been reported in many countries in America, Asia, Africa, and Europe [2]. In China, 14 OPA cases were reported in a farm in Shandong with 400 Dorper sheep, with an estimated morbidity of 3.5% and fatality rate of 100% [3]. Affected animals typically present with clinical features such as respiratory distress, coughing, and emaciation, which are not easily discernible [3]. Consequently, they may be overlooked by livestock owners, allowing infected sheep to continue spreading the virus within the flock. By the time sheep exhibit overt clinical symptoms, extensive lung tumours have developed, necessitating culling or slaughter [4]. Notably, during illness, sheep within the same or different flocks may have become infected. Furthermore, there is currently no effective vaccine for preventing JSRV infection globally, and no specific treatment is available [5]. Therefore, OPA poses a hindrance to the sustainable health development of the global sheep industry.

Several JSRV detection methods have been established globally, including nucleic acid detection methods [6,7,8], double antibody sandwich enzyme-linked immunosorbent assay [9], and recombinase polymerase amplification (RPA) [10]. However, owing to the inability to detect circulating antibodies in sheep with OPA, there is currently no global serological method for detecting JSRV [4]. Additionally, these methods are susceptible to interference from endogenous JSRV, affecting the specificity, sensitivity, and accuracy of established detection methods [11]. The World Organization for Animal Health (WOHA) asserts that there are currently no reliable laboratory methods for diagnosing OPA in live animals and relies on post-mortem examination and pathological diagnosis [12]. The OPA lung exhibits typical pathological changes characterized by the proliferation of type II alveolar epithelial cells, with cuboidal or cylindrical tumour cells replacing normal thin alveolar epithelial cells and sometimes growing into alveoli in a papillary manner. Therefore, the WOHA continues to use pathological diagnosis as the gold standard for diagnosing OPA.

Digital pathology is a novel field in the study of pathology, which greatly enhances the efficiency of personnel involved in pathology by digitizing pathological slides and providing whole-slide images (WSI), thereby improving workflow [13]. Artificial intelligence (AI) is a cutting-edge technology of the 21st century. In recent years, AI has shown great potential in the medical field [14,15,16]; for instance, it has been used in drug discovery and development to predict the therapeutic response to targeted drugs, immune checkpoint inhibitors, and chemotherapy drugs [17]. In addition, AI can extract and classify organizational features from a large number of pathological images and deeply explore the key information within these images to achieve autonomous tumour diagnosis, histological grading, prognosis prediction, and other tasks, leading to precise diagnosis [18,19]. With the rapid development of deep learning (DL) based on convolutional neural networks (CNN), CNN models have been applied to various aspects of veterinary diagnosis, such as the detection of parasites [20], identification of cells [21], and radiology tasks [22,23,24]. Furthermore, CNN has been proven to have the potential to detect and classify tumours [25,26]. CNN-based instance segmentation technology has been rapidly advancing and widely applied in tasks such as human image segmentation and medical image processing. Instance segmentation comprises two stages: (1) Object detection and localization and (2) classifying detected regions based on their features [27]. Currently, the most commonly used model structure for segmentation in medical pathological images is U-Net. However, U-Net lacks a classification subnetwork, imposing certain limitations on its application in multipathological scenarios (lesion localization and classification) [28]. High-performance instance segmentation methods are primarily based on You Only Look Once (YOLO), mask R-CNNs, and their derivatives [28]. Mask R-CNN not only performs object classification and bounding box regression but also generates precise segmentation masks for each object. In contrast, YOLO is renowned for its high prediction speed and low performance. Therefore, the Mask R-CNN model is preferable to YOLO for medical pathology images. Currently, few models based on deep learning have been developed for diagnosing pathological images of animal diseases, and there are no reports on the establishment of OPA pathological image diagnostic models.

In this study, an effective diagnostic model for OPA pathological tissue sections was developed using a Mask R-CNN based on deep learning. Model performance was evaluated using mean average specificity (mASp) and average sensitivity (ASe) and compared with privacy-protected validation and pathologists’ diagnosis results to assess performance in clinical practice. This model aims to assist various personnel in rapidly diagnosing OPA pathological images in practical applications while also laying the groundwork for the transition from traditional histopathology to digital histopathology in the livestock industry.

## 2. Materials and Methods

### 2.1. Pathological Image Sources of OPA

**Figure 1:**
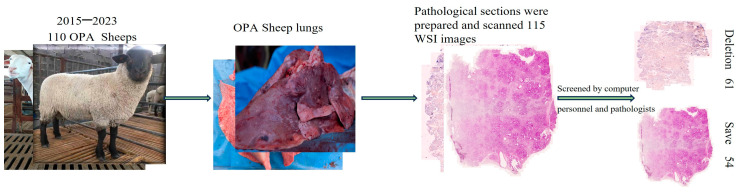
Image source and selection process.

This study included 110 sheep diagnosed with OPA by our laboratory between 2015 and 2023, and 115 histological slides of OPA-positive lung tissues from these cases were collected between 2015 and 2023. The slides were scanned using an automatic digital slide scanner (ZEISS Axio Scan.Z1, Hohhot, China) at a magnification of 40×. Low-quality images owing to extreme fading, low resolution, or abnormal staining, images of the same positive case, and weak pathological changes were excluded. Finally, 54 OPA lung pathological images were selected by pathologists and computer personnel after quality control (Figure 1), and 49 were randomly selected for the construction of the Mask R-CNN OPA pathological diagnostic model training dataset. The remaining five were used for privacy-preserving image validation and evaluation of the actual application performance.

### 2.2. Mask R-CNN Algorithm

**Figure 2:**
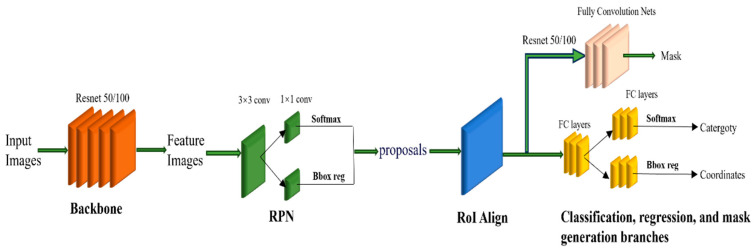
Mask R-CNN architecture.

A mask R-CNN (R-CNN) is a deep learning model that extends object detection to achieve instance segmentation. It comprises four main components: (1) Feature extraction network (backbone): Typically, a deep residual network or feature pyramid network is used as the feature extractor to capture rich features from input images. (2) Region proposal network: Responsible for generating candidate regions (proposals) on a feature map that may contain objects. This is conducted by applying multiple anchors with different scales and aspect ratios using sliding windows and performing binary classification (foreground or background) and bounding box regression for each anchor to generate candidate regions. (3) RoIAlign layer: Fixed-size feature maps corresponding to candidate regions are extracted through bilinear interpolation from the feature map and are used for subsequent classification and mask generation tasks. (4) Classification, regression, and mask generation branches: the classification and regression branches classify candidate regions and perform bounding box regression to obtain more precise object location and category information, while the mask generation branch employs a fully convolutional network to classify each pixel within the candidate region to generate object masks. The output of this branch is a binary mask of the same size as the candidate region, where pixels belonging to objects are labelled as 1 and background pixels are labelled as 0 (Figure 2).

### 2.3. Establishment of the Training Dataset for the Mask R-CNN OPA Pathological Diagnostic Model

**Figure 3:**
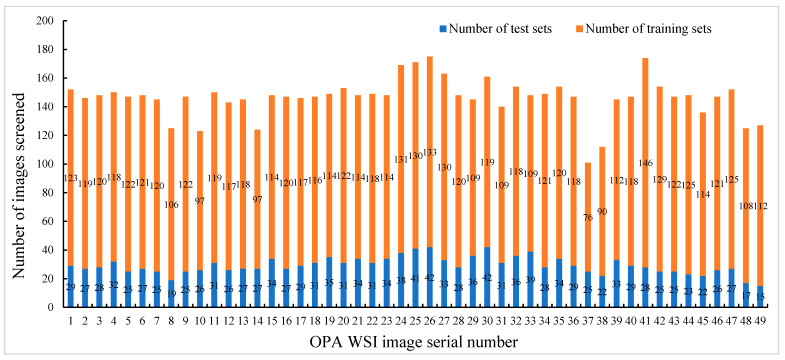
OPA WSI screening.

**Figure 4:**
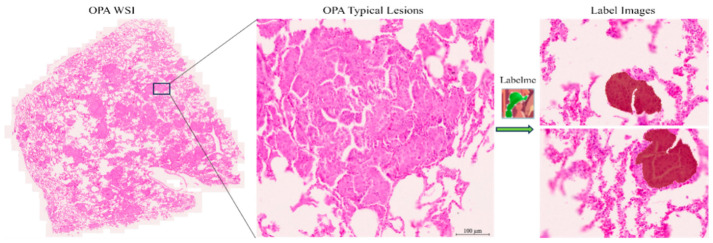
Labelling OPA images with Labelme.

First, our goal was to diagnose OPA on pathological images by identifying and locating OPA lesions. Considering the range of OPA lesions, we used Zen 3.4 software to segment 49 images into 1600 × 1200 pixels. Pathologists screened the segmented images to remove atypical OPA lesions to ensure that each individual image in the dataset was rich enough to effectively support subsequent model training and accurate recognition. Pathologists were required to (1) ensure that the image contains typical OPA lesions; (2) screen the images to ensure that the ratio of the OPA lesion area to the healthy area in each image is approximately 1:1; (3) ensure that the number of segmented images selected in each WSI image is consistent, as far as possible. The number of segmented images screened for each OPA WSI pathological image is presented in Figure 3 (containing the sum of the training and test sets). The screened images were re-screened by other pathologists to ensure the accuracy and consistency of the data. To ensure performance in clinical practice, we compressed the screened pathological images to 800 × 600 pixels using Photoshop 2021. Finally, a pathologist used the Labelme image annotator to mark typical OPA lesions (characterized by severe proliferation of type II alveolar epithelial cells in a cubic or columnar arrangement, with papillary growth extending into alveolar spaces, fusion between alveoli forming large diffuse “cauliflower-like” adenomatous lesions) and atypical lesions and labelled them as OPA (Figure 4). Images were stored as Josn files. After segmentation, screening, elimination, and annotation, we established a Common Objects in Context (COCO) dataset containing 7167 annotated images with 61,063 typical OPA lesions for pathological imaging analysis.

### 2.4. Training of the Mask R-CNN OPA Pathological Diagnostic Model

**Figure 5:**
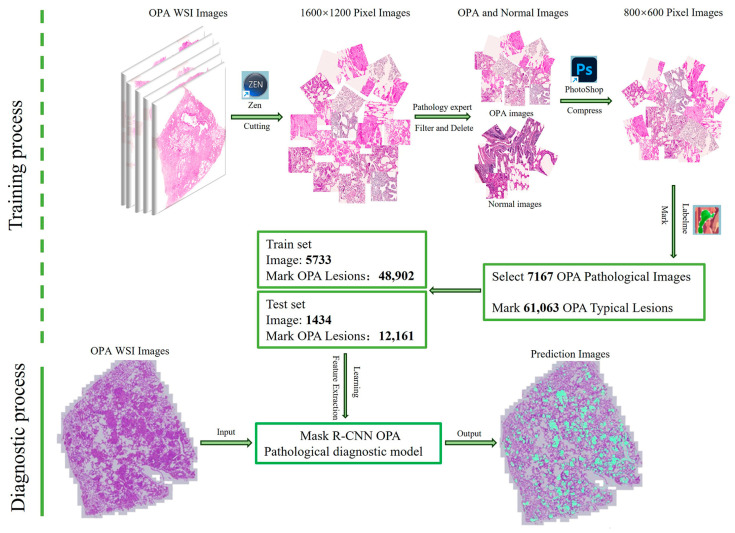
Training process and actual diagnostic process of the Mask R-CNN OPA pathological diagnostic model.

To better simulate the actual diagnostic environment, enhance the generalization ability of the model, and mitigate the impact of overfitting, the Albumentations library was used to randomly augment the images when loading the model training data. This involved a 50% probability of horizontal or vertical flipping of the image, a 30% probability of randomly cropping one-third of the width of one side of the image, a 30% probability of randomly cropping one-third of the height of one side of the image, and a 30% probability of the occurrence of small target occlusions (with a maximum height of 18 pixel, a minimum height of 10 pixel, a maximum width of 18 pixel, and a minimum width of 10 pixel). This was to simulate the diversity of OPA lesions and the complexity of pathological images in actual diagnoses. First, the images were extracted from the Json file. Then, a Python script was used to randomly divide all the extracted images into training and test sets at a ratio of approximately 8:2 (Table 1). The number of training sets and test sets in each WSI image is shown in Figure 3. The dataset was trained on a dedicated Mask R-CNN model, enabling end-to-end diagnosis. During the diagnostic process, pathological images, such as WSI images, were input to the model, which then automatically compressed and segmented them, predicted all segmented images, highlighted the identified OPA lesions, and combined all segmented images to output the WSI image. The training process and actual diagnostic procedure are illustrated in Figure 5.

### 2.5. Performance Evaluation Metrics of Mask R-CNN OPA Pathological Diagnostic Model

We used the mASp metric to assess model performance. mASp was calculated based on the intersection over union (IoU), specificity, and sensitivity values. IoU measures the degree of overlap between the predicted bounding boxes and ground truth boxes, with a typical IoU threshold set at 0.5; if the IoU between a predicted box and a ground truth box exceeds or equals 0.5, it was considered a true positive (TP). Specificity represents the proportion of correctly identified positive samples out of all samples classified as positive by the model, while sensitivity indicates the proportion of correctly identified positive samples out of all actual positive samples. Average specificity (ASp) was computed by calculating the area under the specificity—sensitivity curve for different IoU thresholds, ranging from 0.5 to 0.95 in increments of 0.05 for each image. Subsequently, we obtained mASp by averaging all AP values across images; a higher mASp value signifies greater accuracy in model predictions. In this study, we employed a transformed mASp, where any erroneous prediction within an image results in an assumed mASp value of 0. This transformation allows for a more detailed analysis of error cases to investigate the reasons behind correct and incorrect predictions, and it enables a more rigorous assessment of model performance.

(1) $$IoU=\frac{Area\;of\;Union(P)\backslash cup(G)}{Area\;of\;Overlap(P)\backslash cap(G)}$$

(2) $$specificity=\frac{TP}{TP+FP}$$

(3) $$sensitivity=\frac{TP}{TP+FN}$$

(4) $$mASp=\frac{1}{N}\sum_{i=1}^{N}A{Sp}_{i}$$

False positives (*FPs*) are negative cases that the model incorrectly predicts to be positive cases. False negatives (*FNs*) are positive cases where the model incorrectly predicts to be negative cases.

### 2.6. Anti-Peeking Image Validation

During the training process of deep learning models, the issue of data leakage often arises, wherein the model uses information from the test data during training or when there is a high similarity between the test set and training set data due to their common origin. Such leakage leads to artificially inflated accuracy on the test data but poor performance on unseen data. To mitigate this situation, we conducted an anti-snooping image validation of the Mask R-CNN model. We selected three OPA pathological images and three non-OPA pathological images that were not included in the training set for the independent validation of the Mask R-CNN diagnostic model. This approach effectively assessed the generalizability of the model for new data and ensured its reliability in practical applications.

### 2.7. Evaluation of Mask R-CNN Model Performance in OPA Pathological Image Diagnosis

To further assess the diagnostic performance of the Mask R-CNN model for OPA pathological images, we compared its accuracy, sensitivity, specificity, and concordance rate with those of diagnoses by pathologists. Owing to the limited number of WSI images available, we selected two additional OPA pathological images and three non-OPA pathological images that were not included in the training set. The same pathologist who annotated the OPA lesion used Zen 3.4 software for image segmentation. We prepared 600 OPA pathological images and 1000 non-OPA pathological images. We randomly selected 500 images (200 OPA and 300 non-OPA) at a ratio of approximately 2:3 for analysis. The Mask R-CNN model was used to diagnose these images, classifying them as either OPA or non-OPA based on model annotations. Six pathologists (not including those marking the OPA lesions) from Inner Mongolia Agricultural University’s College of Veterinary Medicine, including three junior pathologists with less than 2 years of experience and three senior pathologists with over 6 years of experience, were invited to participate in this evaluation. Each pathologist independently completed the test, and the average diagnosis was derived for each group. We compared the accuracy, sensitivity, specificity, and concordance rates between the diagnoses made using the Mask R-CNN model and those made by the pathologists to evaluate the model’s performance as an OPA pathology diagnostic tool (Table 2).

## 3. Results

### 3.1. Model Training Environment and Parameters

The platform used in this experiment was a Linux server running Ubuntu Server 22.04 operating system. The hardware configuration of the server comprised two Intel(R) Xeon(R) Gold 6139 M CPU @ 2.30 GHz processors(Intel Corporation, purchased from Hohhot, China), 128 GB of RAM, and seven NVIDIA GeForce RTX 4090 graphics cards(Nvidia Corporation, purchased from Hohhot, China) On the software front, the system environment comprised Python 3.10.11, CUDA 11.7, and the deep-learning framework PyTorch 2.0.1. The learning algorithm employed in this study used stochastic gradient descent (SGD) for optimization with an initial learning rate set at 0.02, a momentum value of 0.9, and a weight decay parameter of 0.0001. Due to memory constraints, a batch size of 96 was selected (refer to Table 3).

### 3.2. Training Results of Mask R-CNN OPA Pathological Diagnostic Model

**Figure 6:**
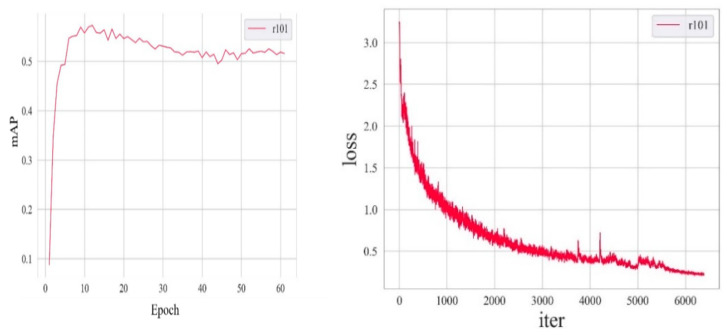
Accuracy and loss mASp of the Mask R-CNN OPA pathological diagnostic model.

**Figure 7:**
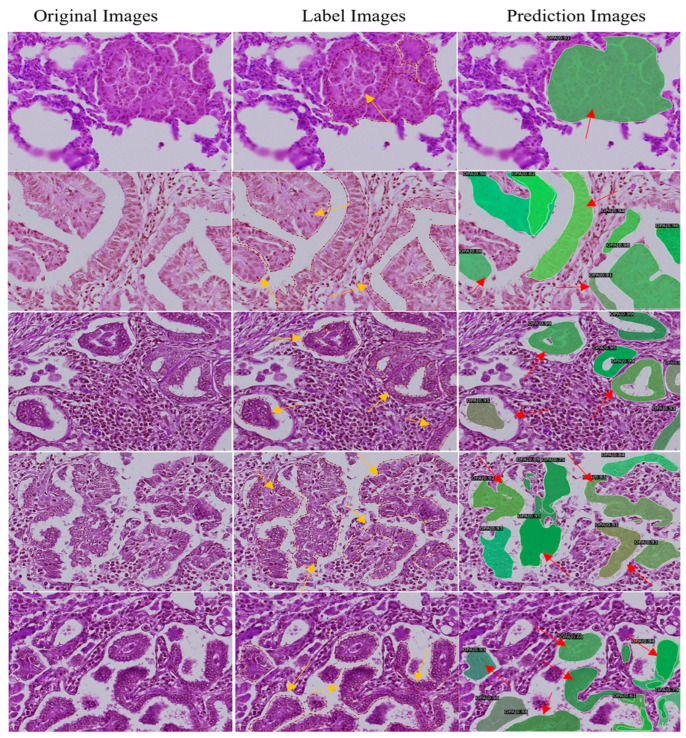
Comparison of original, labelled, and predicted images in the Mask R-CNN OPA pathological diagnostic model. Left panel: original image plot. Middle: labelled images used for OPA lesion identification. Right: image of the lesion area predicted by the model. Yellow arrow points to the OPA lesion marked by the pathologist. Red arrows point to models predicting OPA lesions.

The training results of the Mask R-CNN OPA pathological diagnostic model are presented in Figure 6 and Table 4. At the 12th epoch, the mASp value reached its highest point of 0.573, indicating an accuracy of 57.3% for diagnosing OPA pathology using this model. The loss chart reveals a rapid decrease in the loss values from 0 to 500 iterations, followed by a continuous decline and gradual stabilization between 500 and 6000 iterations, with overall small fluctuations remaining below 0.5. As shown in Table 4, the ASe value gradually increased with each epoch, reaching a peak of 0.745 during the twelfth epoch. Figure 7 shows a comparative analysis of the representative original, annotated, and model-predicted images. This demonstrates that the model effectively distinguishes between lesion areas and normal regions across different images while exhibiting excellent overlap with pathologist-annotated images.

### 3.3. Mask R-CNN OPA Pathological Diagnostic Model Anti-Peeking Verification Results

**Figure 8:**
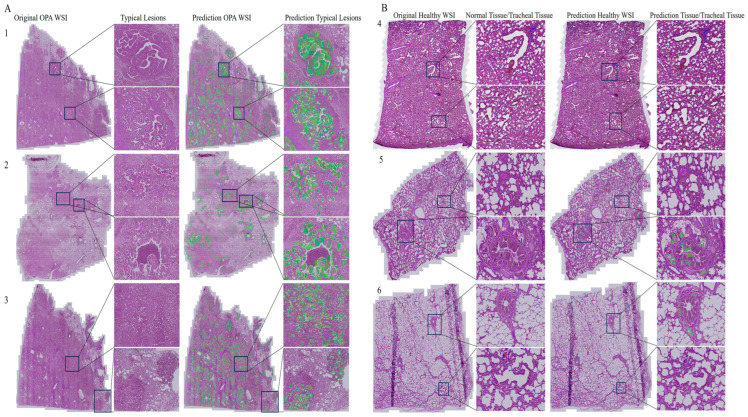
Anti-peeping verification results of the Mask R-CNN OPA pathological diagnostic model Notes: (**A**) OPA WSI diagnostic results; (**B**) non-OPA WSI diagnostic results 1–6 numbers represent WSI pathological pictures prepared from lungs of different sources.

We used six WSI pathological images, including three OPA pathological images and three non-OPA images, which were not included in the dataset used for model training. The model demonstrated excellent capability in identifying OPA lesions within OPA pathological images (Figure 8A). It could specifically recognize and differentiate OPA lesions from non-lesion areas on complex pathological images (Figure 8(A2)). In the non-OPA pathological images, the model also exhibited satisfactory performance (Figure 8B); however, it showed a small number of predicted lesion areas around the small bronchi or fine bronchioles (Figure 8(B5)). Overall, during privacy verification, the model displayed a strong performance in localizing OPA lesions and distinguishing between OPA and non-OPA pathological images.

### 3.4. Comparison of Mask R-CNN OPA Pathological Diagnostic Model with Pathologist Diagnoses

**Figure 9:**
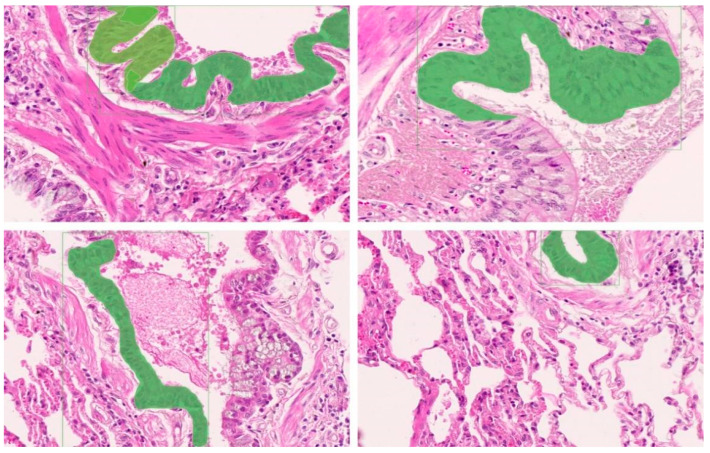
Lesion prediction of the Mask R-CNN OPA pathological diagnostic model in non-OPA images.

In a random sample of 500 images, the Mask R-CNN model accurately diagnosed 200 OPA images and 264 non-OPA images, yielding an accuracy rate of 92.8%, sensitivity of 100%, and specificity of 88% (Table 5). Representative predictions for non-OPA pathological images by the model are illustrated in Figure 9, which shows that the model primarily localized lesions around the bronchial tissue. Junior pathologists achieved an average accurate diagnosis of 172 ± 7.5 OPA images (95% confidence interval [CI]: 163.281–180.719) and 269 ± 4.04 non-OPA images (95% CI: 258.964–279.036), resulting in an accuracy rate of 88.4%, sensitivity of 86%, and specificity of 89.3% (Table 5). Senior pathologists, on average, diagnosed 192 ± 3.05 OPA images (95% CI: 184.423–199.577) and 289 ± 2.64 non-OPA images (95% CI: 282.442–295.558), with an accuracy rate of 96.2%, a sensitivity of 96%, and a specificity of 96.3% (Table 5). As shown in Table 5, the accuracy rate, sensitivity, and specificity of senior pathologists were higher than those of junior pathologists. The Mask R-CNN model demonstrated higher accuracy and sensitivity than junior pathologists, achieving a 100% agreement rate in diagnosing the correct images. Furthermore, its sensitivity surpassed that of senior pathologists, with a 96.5% agreement rate in diagnosing correct images.

## 4. Discussion

In recent years, the application of Mask R-CNN has played an increasing role in the development of pathology diagnostic models, with its robust instance segmentation capability offering significant support for precision medicine [29,30,31]. This research is centred on leveraging Mask R-CNN to construct a diagnostic model specifically targeting OPA, with the aim of enhancing the efficiency and accuracy of actual diagnoses performed by medical professionals.

This study successfully established an OPA pathology diagnostic model based on Mask R-CNN, which exhibited promising performance with an mASp of 0.573 and ASe of 0.745. Wang J et al. used approximately 7500 pathological images from the PanNuke dataset to develop a diagnostic model for cancer tissue pathology using Mask R-CNN. The mASp was 0.269 owing to challenges in the model’s recognition task, such as detecting and classifying each nuclear cell in every pathological image [32]. Although Mask R-CNN can accurately locate the detection target, challenges remain in the actual diagnosis of complex pathological images, for example, the lesion area in medical images often has blurred boundaries and an irregular shape. Freitas et al. refined object boundaries and improved segmentation accuracy by introducing the Multiple Mask and Boundary Scoring R-CNN (MM&BS R-CNN) in the classification of transurethral resection of bladder tumour. The mASp value of the model was increased by 17.44% to 0.496 [28]. In our study, we leveraged data augmentation to assist the model in learning more complex boundary scenarios and employed OPA pathological images from an independent dataset to ensure the model’s generalization capability across different datasets, aiming to enhance our model’s mASp value. Nevertheless, model recognition is complicated by the complexity of the recognition task, including the diversity of OPA lesions and the need to identify each hyperplastic cell contributing to the OPA lesion, coupled with the challenges posed by pathological images in which lesion and non-lesion areas do not have distinct boundaries within the same image. Overall, the accuracy of the model was satisfactory. To visually demonstrate the model’s accuracy in identifying OPA lesions, we compared pathologist-annotated images with model predictions for different image patches segmented from WSI. The annotation maps and prediction maps showed good overlap. Baek et al. established a diagnostic model to segment drug-induced liver injury and compared the predicted results of the model with ground truth annotations generated by certified toxicological pathologists. Similarly, the labelled map and the predicted map showed good overlap [33]. This advance demonstrates the reliability of our model for the diagnosis of OPA pathology. Overall, the above results collectively indicate that the Mask R-CNN OPA pathological diagnostic model is a viable tool for diagnosing OPA pathological images.

Peeking, characterized by high accuracy in test data but erroneous results in unseen data, is one of the most challenging problems encountered by deep-learning scientists. Peeking is related to overfitting [34]. In developing the diagnostic model for feline hypertrophic cardiomyopathy, Rho et al. prevented the occurrence of peeking by strictly dividing the training data set and the validation data set, as well as testing the model on a new data set. Despite these measures, the model still showed signs of peeking [34]. In this study, to prevent peeking and to mitigate overfitting, we employed two approaches. First, the dataset was explicitly divided into a training set and a test set. Second, to better simulate the actual environment and enhance the generalization ability of the model, we utilized the Albumentations library to randomly augment the images when loading the training data for the model. This resulted in a 50% probability of the image being horizontally or vertically flipped and a 30% probability of one-third of the width on one side of the image being randomly cropped. Additionally, by evaluating the anti-peeking ability of the Mask R-CNN OPA pathology diagnostic model using images that have never been used in the training and testing process (including three OPA pathological images and three non-OPA images), we further demonstrated the model’s ability to locate OPA lesions and diagnose OPA pathology in the real world, rather than based on a dataset. The Mask R-CNN OPA pathology diagnostic model developed in this study did not avoid the phenomenon of peeking. Nevertheless, the model remains capable of specifically identifying and differentiating OPA lesion areas from non-lesion areas in complex pathological images. However, in non-OPA images, there are some predicted lesion regions around the bronchi or fine bronchi. This can be mainly attributed to the high similarity between the morphology of the pseudostratified columnar epithelium of small bronchi or single-layered columnar epithelium of fine bronchi and that of OPA lesions. This significantly affects the model’s ability to recognize actual OPA lesions but does not impede its utility in assisting junior or experienced pathologists in diagnosing WSI-level OPA pathological images. Overall, the anti-peeking verification results demonstrate excellent performance of the model in locating OPA lesions and diagnosing OPA pathological images in clinical practice.

Traditional pathological diagnosis relies on the observation of HE-stained tissue sections under microscopy. Difficulties arise in diagnosis and analysis due to the complexity of pathological image information, making the process time-consuming and laborious. Furthermore, the diagnosis of pathological images is highly dependent on the subjective evaluation and experience of the pathologist. We compared the model’s results with the diagnostic results of pathologists to assess whether the model can effectively assist pathologists in achieving rapid and accurate OPA diagnosis in clinical settings. The Mask R-CNN OPA pathology diagnostic model performed well, with an accuracy rate falling between that of junior and senior pathologists (88.2% and 96.2%, respectively). Notably, the model achieved a 100% agreement rate with junior pathologists and a remarkable 96.5% agreement rate with senior pathologists, demonstrating its significant potential as an auxiliary diagnostic tool. Similarly, Hwang JH et al. compared Mask R-CNN model predictions with diagnoses made by pathologists, demonstrating the effectiveness of the model in diagnosing liver fibrosis in actual diagnostic scenarios [29]. Furthermore, the authors found that the subjective element of diagnosis by pathologists and variations in diagnostic proficiency led to differences in final accuracy [29]. This is consistent with the findings in this study, in which junior and senior pathologists demonstrated different specificity, sensitivity, and accuracy rates (88.2%, 86%, 89.6% and 96.2%, 96%, 96.3%, respectively). Fragoso-Garcia et al. argue that the reason for diagnostic discrepancy between automated diagnosis and diagnosis by pathologists lies in cognitive or psychological factors. Once pathologists make an initial diagnosis, they may exhibit overconfidence, potentially overlooking suspicious lesions during the diagnostic process, whereas model diagnoses are objective [26]. In the present study, junior and senior pathologists misidentified 18 and 8 OPA images, respectively. Pathologists initially diagnose OPA images by identifying OPA lesions, and when evaluating the lesions, they consider multiple aspects including the cells, shapes, and locations that constitute the lesions. Image blocks merely contain the pseudostratified columnar epithelium of some small bronchi or the monolayer columnar epithelium of bronchioles, thereby resulting in the misjudgement of OPA images by pathologists. The result of model identification is identical to the anti-peeking verification result, being influenced by the pseudostratified columnar epithelium of small bronchi or the monolayer columnar epithelium of bronchioles. This further suggests that variations in image quality, such as incomplete representation of certain tissues or differences in staining, can also affect the assessment of pathologists. Furthermore, it highlights the importance of distinguishing between the pseudostratified columnar epithelium of small bronchi or the simple columnar epithelium of bronchioles and OPA lesions, which is of great significance for the development of OPA pathology diagnostic models. In conclusion, the abovementioned results demonstrate the capability of this model to localize lesions and diagnose OPA lesions within OPA pathological images, suggesting that this model can assist pathologists in diagnosing OPA.

The successful development of this model demonstrates the considerable potential of artificial intelligence-based diagnostic models in the fields of animal husbandry and veterinary medicine and lays a foundation for the further development of AI-powered pathology diagnostic models in these areas. Nevertheless, some limitations of the present study should be noted. The dataset used for the actual WSI OPA pathological image construction was limited to 49 images. Despite the meticulous labelling of both typical and atypical OPA lesions within the images, it is plausible that there exist OPA lesions that the model remains incapable of identifying during actual diagnostic scenarios, thereby exerting a certain degree of influence on the model’s overall performance. Furthermore, the annotations within the dataset were provided by a single pathologist and, as such, were potentially influenced by their professional expertise. In the future, as an increasing number of OPA disease cases are identified, our research group aims to expand both the quantity of OPA pathological images and enhance annotation methods. This will improve the accuracy of the Mask R-CNN OPA pathological diagnostic model.

## 5. Conclusions

This study successfully created a COCO dataset of OPA pathological images and developed an OPA pathological diagnostic model based on Mask R-CNN. The model achieved a maximum mASp value of 0.573 and an ASe of 0.745. In practical applications, the model demonstrated accuracy of 92.8%, sensitivity of 100%, and specificity of 88%. The agreement rates between junior and senior pathologists were 100% and 96.5%, respectively. In conclusion, the successful development of this model facilitates rapid OPA diagnosis in practical applications and lays a foundation for the transition from traditional to digital pathology in the livestock industry.

## Figures and Tables

**Table 1:** Training and test set information.

	Image	Label
Training Set	5733	48,902
Test Set	1434	12,161

**Table 2:** Evaluation criteria.

Project	Method
Accuracy	Correct identification number/All images
Sensitivity	Correctly identify OPA/Real OPA
Specificity	Correctly identify non-OPA/Real non-OPA
Compliance rate	Mask R-CNN Model recognition correct identification number/Pathologist recognition correct identification number

**Table 3:** Hyperparameters used for training Mask R-CNN algorithm.

Hyperparameters	Value
Learning_ Rate	0.02
Learning_ Momentum	0.9
Weight_ Decay	0.0001
Batch_ Size	96
Max_ Epochs	65
Warm up learning rate schedule	LinearLR(Epoch 1–5)
Main learning rate scheduler	LinearLR(Epoch 6–65)

**Table 4:** mASp and ASe of the Mask R-CNN OPA pathological diagnostic model.

Epoch	mASp	ASe
1	0.087	0.415
2	0.347	0.586
3	0.455	0.647
4	0.492	0.666
5	0.493	0.668
6	0.547	0.734
7	0.551	0.736
8	0.552	0.722
9	0.569	0.744
10	0.557	0.737
11	0.569	0.744
12	0.573	0.745
13	0.558	0.723
14	0.557	0.727
15	0.564	0.740

Note: epoch: number of learnings; mASp: mean average specificity; ASe: average sensitivity.

**Table 5:** Comparison between the Mask R-CNN OPA pathological diagnostic model and pathologists.

	Correctly Identify OPA/Real OPA	Correctly Identify Non-OPA/Real Non-OPA	Accuracy	Sensitivity	Specificity	Compliance Rate
Mask R-CNN model	200/200	264/300	92.8%	100%	88%	
Junior Pathologist	172 ± 3.51/200	269 ± 4.04/300	88.2%	86%	89.6%	100%
Senior Pathologist	192 ± 3.05/200	289 ± 2.64/300	96.2%	96%	96.3%	96.5%

## Data Availability

The datasets analyzed in this study are available from the corresponding author on reasonable request.
